# A case of complex balanced chromosomal translocations associated with adverse pregnancy outcomes

**DOI:** 10.1186/s13039-022-00615-z

**Published:** 2022-08-21

**Authors:** Yan Luo, Hezhen Lu, Yanshang Zhang, Zhiqiang Cui, Pingping Zhang, Yali Li

**Affiliations:** 1 Department of Reproductive genetics, Hebei General Hospital, No. 348 Heping West Road, Xinhua District, Shijiazhuang, 050051 Hebei China; 2 Department of Obstetrics, Hebei General Hospital, Shijiazhuang, 050051 Hebei China; 3 Department of Breast Surger, Affiliated Hospital of Hebei Engineering University, Handan, 056004 Hebei China

**Keywords:** Complex chromosomal rearrangements, Chromosomal abnormality, CCR carrier

## Abstract

Complex chromosomal rearrangements (CCR) are rare chromosomal structural abnormalities. The chromosomal structural variants in CCR carriers are one of the factors contributing to a history of adverse pregnancy and childbirth. In this study, we report a patient with a history of adverse pregnancy and childbirth who exhibited complex balanced chromosomal translocations. The female patient was phenotypically and intellectually normal; in her first pregnancy, the embryo was damaged, and a histological examination of the chromosomes of the embryos revealed a deletion of approximately 4.66 Mb at 1p32.3p32.2, a duplication of approximately 1.02 Mb at 1p22.2p22.1, a duplication of approximately 1.46 Mb at 6q27 and a deletion of approximately 7.78 Mb at 9p24.3p24.1. Chromosomal examinations of the patient revealed the karyotype to be 46,XX,(1;9)(p32; p34). In the second pregnancy, the foetus was diagnosed prenatally with three or more positive ultrasound soft indicators. The patient's karyotype was re-examined and further confirmed by fluorescence in situ hybridisation as 46,XX,t(1;9;6)(p31;p22;q27), revealing this patient was a carrier of complex balanced chromosomal translocations. Carriers of CCR have a higher risk of spontaneous abortion, and genetic counselling clinicians should consider the karyotype analyses of such patients in clinical practice and recheck their chromosomes if necessary.

## Preface

Complex chromosomal rearrangement (CCR) is a rare structural chromosomal abnormality characterised by three or more breakpoints on two or more chromosomes accompanied by inter- or intra-chromosomal single-segment insertions or translocations, etc. [1–3]. As a rare structural recombination, CCR can be balanced or unbalanced [4, 5]. Individuals carrying CCR can be phenotypically normal or exhibit clinical abnormalities [6]. The degree of clinical abnormality in congenital malformations or intellectual dysfunctions in carriers of CCR ranges from normal to mild to severe [7]. Such clinical abnormalities are caused by microdeletions or microduplications near translocation breakpoints, gene disruptions and positional effects located at breakpoints or elsewhere in the genome [8]. Additionally, the likelihood of phenotypic abnormalities increases with the number of CCR-associated breakpoints [3].

Adverse pregnancy outcomes refer to the history of spontaneous miscarriage, *embryonic arrest*, foetal death, stillbirth, neonatal death and the birth of mentally impaired and malformed babies [9]. There are many causes of adverse pregnancy outcomes, including genetic, anatomical, immunological and biological factors [10–12]. Chromosomal abnormalities in parents or embryos are the most common cause of adverse pregnancy and childbirth history [13–15]. A statistical analysis of previous studies revealed that 3.5% of couples with a history of recurrent miscarriage have at least one partner who is a CCR carrier [16]. The most frequent type of CCR is translocations, while other rearrangement types include inversions, insertions, deletions and duplications. Additionally, it was found that about 18.4% of CCR carriers produce phenotypically abnormal offspring [16]. Thus, it is of great importance to analyse as accurately as possible whether patients with a history of adverse pregnancies are CCR carriers and to assess the risks they face.

Along with the development of molecular cytogenetics, techniques such as Giemsa banding (G-Banding), fluorescence in situ hybridisation (FISH) and copy number variation sequencing (CNV-seq) have been applied to study chromosomal structural changes [17], and hidden and complex chromosomal rearrangements are yet to be revealed. In this paper, we report the case of an adult female patient with a normal phenotype and intelligence and a history of adverse pregnancy and delivery who was found to be a carrier of complex balanced chromosomal translocations.

## Materials and methods

### Clinical data of study subjects

The patient was a 22-year-old female, with a normal phenotype and intelligence. She was 158 cm tall and weighed 48 kg. The patient and her husband were nonconsanguineous, and the patient had had two post-marital pregnancies and one embryonic arrest in 2020, with a chromosomal examination via embryonic histology. During the second pregnancy (20 weeks + 3 days), the patient requested a prenatal diagnosis at the Department of Reproductive Genetics at Hebei Provincial People's Hospital. The study was approved by the institutional ethics committee for sample collection, and the patient provided signed informed consent.

### Cytogenetic analysis of dominant bands

The patient underwent a metaphase chromosome analysis by G-Banding at a resolution level of 400 bands, and karyotyping was performed on her husband. The karyotype description was based on the recommendations of the International System for Human Cytogenetic or Cytogenomic Nomenclature [18].

### Molecular cytogenetic analysis

A combination of Tel1p (green), Tel1q (red), CEP1 (red), Tel6q (red), CEP6 (white), Tel9p (green) and CEP9 (white) probes were used during FISH for high-resolution molecular cytogenetic analysis.

### Chromosome karyotype analysis

The cell division phase was first observed under a 10 × microscope before being transferred to a 100 × oil immersion lens for detailed observation. Thirty division phases were counted, and those with abnormalities were doubled for counting and analysis. Three of the division phases with appropriate length, clear bands and good dispersion were analysed and diagnosed, and karyotype maps were drawn and printed. The karyotype description was based on the recommendations of the International System for Human Cytogenetic Nomenclature [18].

## Results

In 2020, the patient experienced *embryonic suspension*, and chromosomal examination via embryonic histology revealed a deletion of about 4.66 Mb at 1p32.3p32.2, a duplication of about 1.02 Mb at 1p22.2p22.1, a duplication of about 1.46 Mb at 6q27 and a deletion of about 7.78 Mb at 9p24.3p24.1. The chromosomes of both husband and wife were examined, and the husband’s chromosomes were normal. However, the chromosomal examination of the female partner showed 46,XX,t(1;9)(p32;p34), i.e. a translocation of short arm 3 of chromosome 1 at band 2 to short arm 3 of chromosome 9 at band 4.

The patient came to our department for prenatal diagnosis in her second pregnancy at 20 weeks + 3 days of gestation. She denied any history of exposure to toxic or harmful substances or radiation during her pregnancy. Foetal ultrasonography was performed in our department and indicated that the foetal cerebellum was slightly small, with a strong light spot in the left ventricle, enhanced echogenicity in both kidneys and localised echogenicity in the lower abdomen. Amniocentesis and amniotic fluid karyotype analyses were performed, and the results showed no numerical or structural chromosomal abnormalities. However, the amniotic fluid CNV-seq revealed a deletion of about 1.6 Mb in the 6q27q27 region (a pathogenic variant) and a duplication of 4.68 Mb in the 1p32.3p32.2 region (a suspected pathogenic variant).

**Fig. 1:**
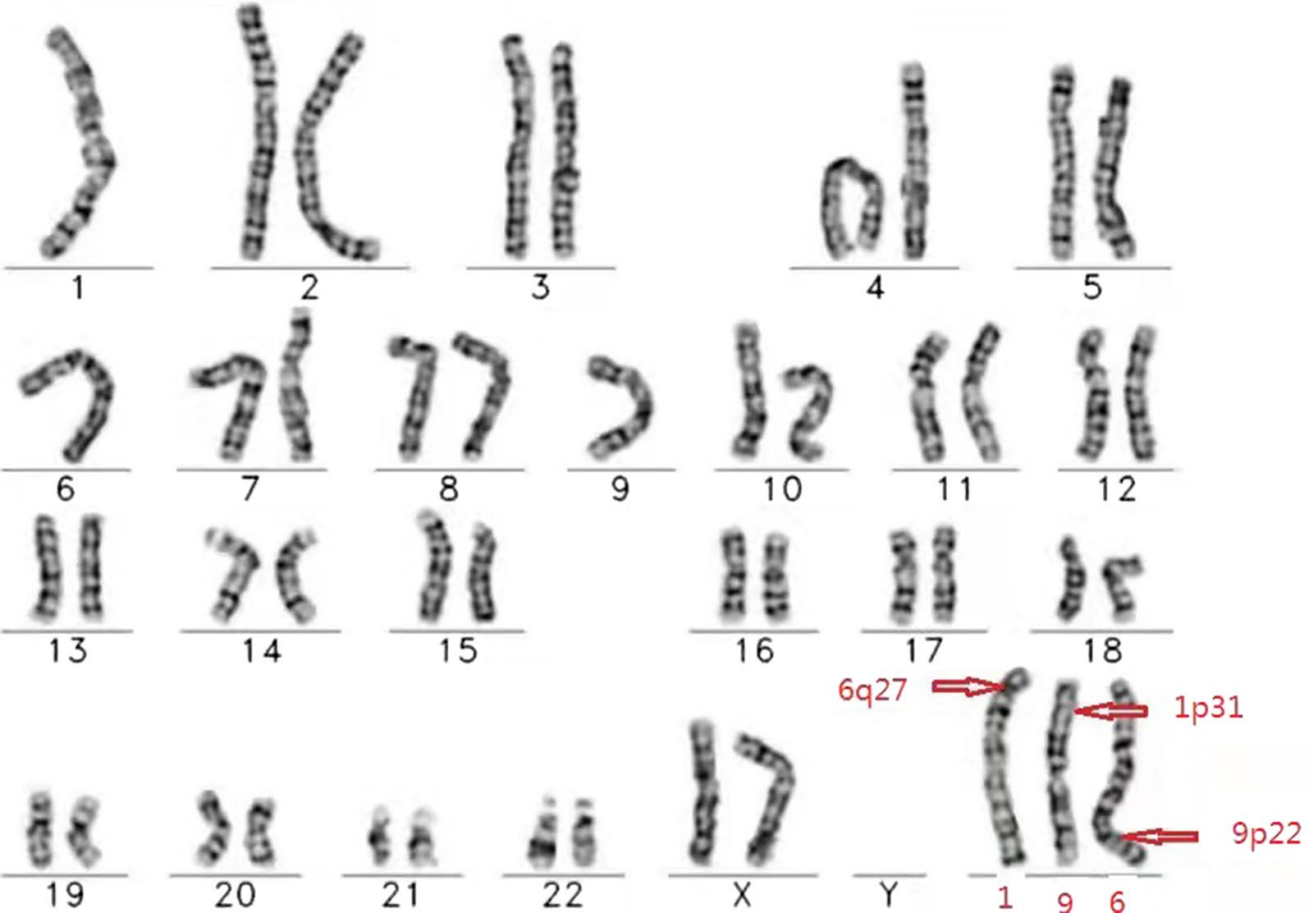
Patient karyotype map: 46,XX,t(1;9;6)(p31;p22;q27)

**Fig. 2:**
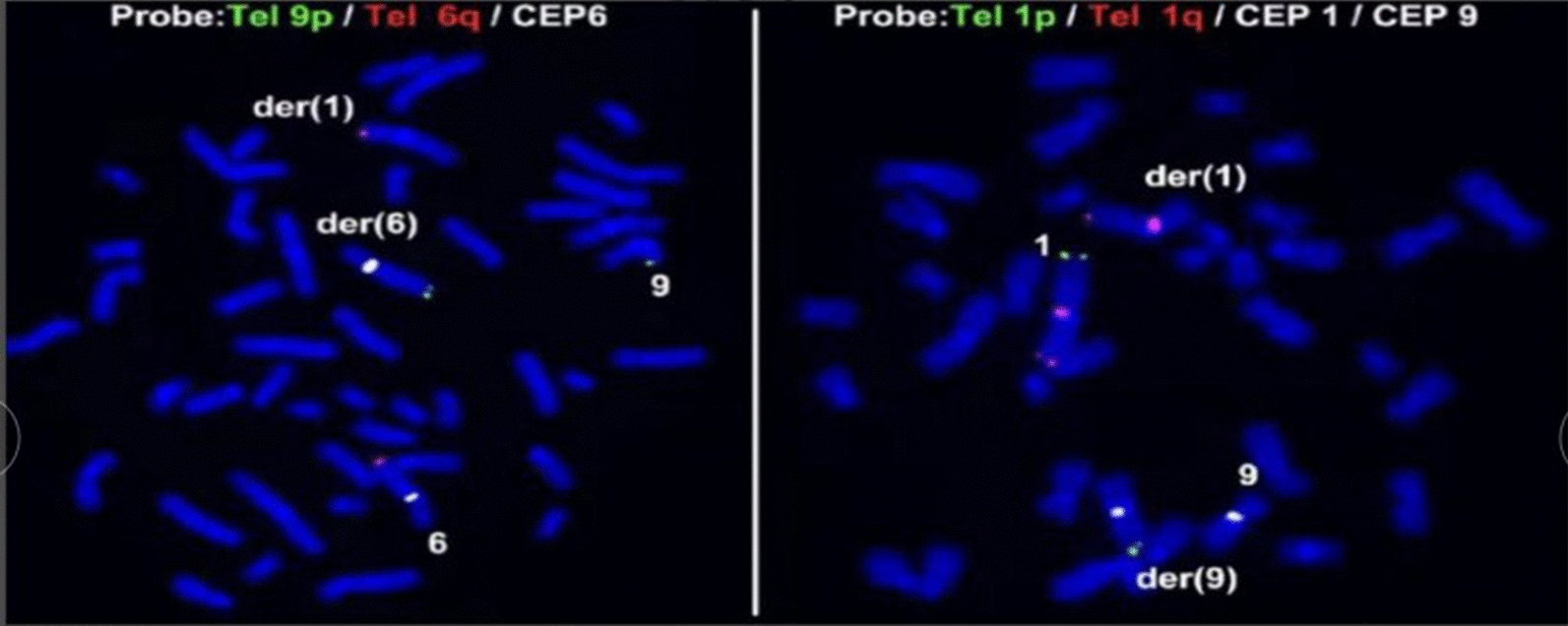
Confirmation of fluorescence in situ hybridization (FISH) results Patient karyotype was 46,XX,t(1;9;6)(p31;p22;q27). *Note*: Figure on the left shows: A Tel 9p (green)/Tel 6q (red) /CEP6 (white) probe combination was applied for FISH with the peripheral blood metaphases of the subject. Thirty division phases were observed, and one derived chromosome 6 and one derived chromosome 1 were observed in each division phase. Figure on the right shows: A Tel 1p (green)/Tel 1q (red)/CEP1 (red)/CEP9 (white) probe combination was applied for FISH with the peripheral blood metaphases of the subject. Thirty division phases were observed, and one derived chromosome 1 and one derived chromosome 9 were observed in each division phase

Given the presence of approximately 1.46 Mb of duplication of chromosome 6q27 in the embryo examined at the time of foetal arrest in the first pregnancy and the results of the amniotic fluid in the second pregnancy, which suggested a deletion of approximately 1.6 Mb in the 6q27q27 region, the female's chromosome was re-examined at the genetics centre of the Reproductive and Genetic Hospital of CITIC Xiangya. The karyotype analysis revealed 46,XX,t(1;9;6)(p31;p22;q27), and a balanced translocation of complex chromosomes 1, 9 and 6 was observed in this patient (Fig. 1). The results were confirmed by FISH. A Tel 9p (green)/Tel 6q (red) /CEP6 (white) probe combination was applied for FISH using the peripheral blood metaphases of the subject. Thirty division phases were observed, and one derived chromosome 6 and one derived chromosome 1 were observed in each division phase (Fig. 2A). The probe combination of Tel 1p (green)/Tel 1q (red)/CEP1 (red)/CEP9 (white) was applied for FISH using the peripheral blood metaphases of the subject. Thirty division phases were observed, and one derived chromosome 1 and one derived chromosome 9 were observed in each division phase (Fig. 2B). The female was reconfirmed via FISH as a carrier of complex translocations of chromosomes 1, 6 and 9.

## Discussion

In the general population, CCR is rarely found. The condition is usually associated with congenital anomalies, mental impairment, recurrent spontaneous abortions and infertility [19]. For example, Sinkar and Devi [20] reported a boy who inherited a significantly balanced chromosomal translocation from his deaf–mute mother, showing mental impairment and aphasia. However, Campos et al. [21] showed that the frequency of balanced CCR in the population may be underestimated since it may not cause phenotypic effects and may not be detected by the analysis method used. This is similar to the results of the present study. The CCR patient reported herein had normal phenotyping and intelligence, but the karyotype analysis revealed that the patient was a complex chromosome translocation carrier.

There are four types of CCRs based on the combination of chromosome number and breakpoints. Type I, the simplest and most common, is inherited maternally; it is characterised by an equal number of chromosomes and breakpoints in CCRs, known as *triple rearrangements* (three chromosomes with one breakpoint on each chromosome), and it is the most common of all CCR cases [22]. Balanced translocations are partly inherited from the parents and partly caused by chromosomes breaking and rejoining during sperm or egg formation or fertilisation of the ovum [23, 24]. During this process, two chromosomes are exchanged after the break, and no increase or decrease of chromosomal fragments is involved. The carrier has normal intelligence and phenotyping, and the clinical manifestations may include infertility, recurrent miscarriages, embryonic arrest and embryonic developmental malformations [25].

Carriers of balanced translocations of two chromosomes form quadriradial chromosomes during meiosis; they produce 18 gametes, of which 1 is normal and 1 is a balanced translocation carrier, with the remaining 16 being unbalanced gametes [26]. The balanced translocations carried by the patient reported in this study involved a total of three chromosomes (1, 9 and 6). They were a carrier of complex translocations, which had an increased chance of chromosomal rearrangement compared with carriers involving only two chromosomes in reciprocal translocations, which are more likely to form unbalanced gametes. These complex balanced translocations were responsible for a history of adverse pregnancy and delivery [27]. In the patient's first pregnancy with foetal arrest, the embryo was examined for chromosomes 1, 6 and 9, with varying degrees of microdeletions or microduplications, and an examination of the patient's chromosomes suggested balanced translocations of chromosomes 1 and 9. In the patient's second pregnancy, the foetus exhibited three or more positive ultrasound soft indicators, which suggested an increased risk of chromosomal foetal abnormalities. The amniotic fluid CNV suggested chromosome 6 microdeletion as a pathogenic variant and chromosome 1 microduplication as a suspected pathogenic variant; both embryonic tissue and amniotic fluid suggested chromosome 6 abnormality, so the patient's chromosomes were re-examined. Finally, the patient was identified as a complex translocation carrier of chromosomes 1, 9 and 6.

The patient's gestational amniotic fluid CNV detected a deletion of approximately 1.60 Mb in the 6q27q27 region, which was a pathogenic variant containing 11 protein-coding genes, including *DLL1*, *THBS2*, *ERMARD* and *TBP*. The *DLL1* gene was evaluated by ClinGen as a single-dose-sensitive gene [28]. *DLL1* acts as a ligand for Notch and plays an important role in Notch signalling [29]. Studies have shown that abnormalities in the *DLL1*/Notch signalling pathway can lead to abnormal embryonic development, dysregulation of biological processes and malignant transformation [30]. In mammalian cells, the *DLL1*/Notch signalling pathway is associated with the maintenance of homeostasis in stem cells [31]^.^ In addition, when Notch signalling is active, the Notch ligand (*DLL1*) binds to Notch receptors on the surface of neighbouring cells where it induces the expression of genes that inhibit neural differentiation, thereby maintaining the cell in a proliferative state. The pathogenic variant formed by the microdeletion of the 6q27q27 region detected by the CNV of this patient's pregnancy amniotic fluid may have been related to the loss of function of the protein encoded by this region of the gene.

In the present study, the patient had discrepancies in the results of two karyotypic analyses; the first showed translocations only of chromosomes 1 and 9, while the second showed complex balanced translocations of chromosomes 1, 6 and 9, which were further confirmed by FISH. There are various reasons for the discrepancy in the results of the two examinations, for example, the resolution (high vs low) of the G-Banding karyotype analysis may have led to variations in the results.

## Conclusion

Carriers of CCR have a higher risk of having both spontaneous miscarriage and offspring with unbalanced karyotypes. Therefore, in clinical practice, genetic counsellors should accurately analyse whether patients with a history of adverse pregnancies are CCR carriers; furthermore, they should evaluate their risks and provide appropriate fertility advice.

## Data Availability

All data generated or analyzed during this study are included in this published article.
